# Editorial: Remote monitoring technologies in neurodegenerative movement disorders

**DOI:** 10.3389/fneur.2024.1421454

**Published:** 2024-07-11

**Authors:** Genko Oyama, Konstantinos Tsamis, Spyridon Konitsiotis, Leonard Verhagen Metman

**Affiliations:** ^1^Department of Neurology and Stroke Unit, Saitama Medical University, Saitama, Japan; ^2^Laboratory of Physiology, Faculty of Medicine, School of Health Sciences, University of Ioannina, Ioannina, Greece; ^3^Department of Neurology, University Hospital of Ioannina and Faculty of Medicine, School of Health Sciences, University of Ioannina, Ioannina, Greece; ^4^Division of Parkinson's Disease and Movement Disorders Center, DBS and Advanced Therapeutics Program, Northwestern University Feinberg School of Medicine, Chicago, IL, United States

**Keywords:** wearable sensors, telemedicine, monitoring, artificial intelligence, digital biomarkers

Neurodegenerative movement disorders, such as Parkinson's disease (PD) and Huntington's disease (HD), pose lifelong challenges to patients. These diseases can significantly affect the quality of life because of progressive impairment of motor control. In addition, the symptoms of these movement disorders vary from individual to individual, and their day-to-day variability leads to difficulties in objective and consistent assessments. In addition, the progressive nature of these diseases often limits a patient's mobility over time, making regular hospital visits even more difficult. Consequently, proper monitoring of symptom progression is a major challenge for healthcare providers, which hampers implementation of appropriate therapeutic strategies. Advances in remote-monitoring technologies have offered innovative solutions to these challenges. Next-generation technology is expected to make it both easier and more precise to track and manage the symptoms of patients with movement disorders, thereby improving their quality of life.

Recent technological innovations have led to revolutionary advances in the management of neurodegenerative disorders. Continuous and accurate patient monitoring is now possible, allowing for the tracking of symptoms in the context of daily life. Automated speech analysis and natural language processing technologies have the potential to be applied to diagnose, evaluate, and monitor diseases by building machine learning models from speech features, based on speech and language changes in each individual patient. Subtle facial movements can be captured from facial expression data and signs of symptom progression, such as changes in emotional expressions, can be evaluated. These are particularly important indicators in patients with PD (1).

Movement data from wearable devices, such as accelerometers and gyroscopes, precisely track a patient's movement patterns (2). This allows for real-time collection and analysis of data on various physical activities, such as gait speed, posture, and balance. The latest non-contact sensors can monitor activities within a living space without the patient having to touch the device directly (3). This technology can capture the natural activities of daily living of a patient and provide continuous data with minimal intrusion (4, 5).

Combining, these technologies could theoretically provide 24/7 insight into the condition of patients with movement disorders in a real-life context, and advances in artificial intelligence (AI) and data analytics could extract useful insights from these vast amounts of data to support the development of treatment plans optimized for individual patients (6).

The use of smartphone applications and teleconsultation may increase adherence to rehabilitation at home. Pastana Ramos et al. examined the safety, feasibility and efficacy of a remote individual rehabilitation program for people with PD living in the Brazilian Amazon. In a randomized controlled trial they compared a telerehabilitation program with a booklet-based exercise program and found high degrees of safety and adherence, as well as improvements in the timed-up-and-go test. Putzolu et al. tested the feasibility, usability and treatment effects of a home-based exercise training program in PD, using a smartphone app. Their results showed high adherence to the training program, high usability and satisfaction, and improvements in PD severity, mobility and cognition. The authors suggested that use of a mobile app may increase physical activity in this patient group. Nunes, Pawlik et al. compared speech in patients with HD, prodromal HD, and normal volunteers.They collected audio recordings to automatically extract speech features, such as pitch, pausing, and accuracy. Analysis with the speech software suggested that speech data may be able to measure HD progression, enable frequent, remote assessment, and potentially serve as marker of clinical onset and disease progression in clinical trials. In a second article, Nunes, Yildiz Potter et al. used accelerometer data from post-stroke patients performing ADLs in order to develop an automated deep learning approach to detect goal-directed movements. Their results suggest that this method may provide a strategy to monitor upper limb movement and provide a biomarker to study upper limb function in neurological disorders. In addition, continuous home monitoring may more accurately detect symptom fluctuations and disease progression. Kamo et al. used wearable devices to measure multiple biomarkers in PD patients and found that the combination of pulse rate and activity index may be a useful indicator of wearing off fluctuations and dyskinetic movements.

Further advances in technology have the potential to improve the quality of life for patients with neurodegenerative movement disorders. Remote monitoring technology will support patients' independence in their daily lives and provide a foundation for more effective symptom management, and ultimately enabling a more fulfilling daily life. AI technology are expected to facilitate the development of personalized medicine and, offering optimal treatment strategies tailored to each patient.

However, plenty of unresolved issues are still associated with these technological advances. These include data privacy and security issues, cost issues, and the difficulty of customization in providing uniformly effective solutions for diverse patient populations. In addition, the development of easy-to-use and intuitive interfaces is essential for the widespread acceptance of these technologies by patients and healthcare providers (7).

In the future, sustained research and innovation in healthcare technology will become more important than ever. We must continue to push the boundaries of technology and strive to provide the best possible support to every patient with movement disorders.

## Author contributions

GO: Writing – original draft, Writing – review & editing. KT: Writing – original draft, Writing – review & editing. SK: Writing – review & editing, Writing – original draft. LM: Writing – review & editing, Writing – original draft.
